# In trauma, when used in the emergency department, do viscoelastic hemostatic tests decrease mortality? A systematic review

**DOI:** 10.1186/cc14422

**Published:** 2015-03-16

**Authors:** J Cousineau, R Daoust, K Doyon, M Marquis, É Piette, JM Chauny, M Clar, D Rose, É Notebaert

**Affiliations:** 1 Hôpital du Sacré Coeur de Montréal, Montreal, QC, Canada

## Introduction

This systematic review done in March and updated in November 2014 has been conducted in accordance with the STARD, PRISMA and STROBE recommendations. Retrospective and prospective studies with a comparison group published in English were kept.

## Methods

Two reviewers (JC and ÉN) and two librarians (MC and DR) independently conducted a systematic review and identified abstracts. Full texts were read by two authors (ÉN and ÉP), and data were extracted. The following databases were searched: Cochrane CENTRAL, Medline, Embase, LILACS, Web of Science, http://Science.gov, SciFinder Scholar, WorldCat, the Transf Evid Lib Database, and proceedings of the congresses of the International Society on Thrombosis and Haemostosis and the American Society of Hematology.

## Results

We initially kept 2,870 references. In total, 453 articles had mortality in their keywords. After reading the abstracts, 37 papers were analysed, and three articles evaluating mortality in two groups of patients (using and not using a VHT) were identified. Among these three studies, only one had raw data available. We did not succeed in getting this information from the two other authors. We asked the main authors of the 37 selected papers, and renowned authors in the field, if they had studies with new data that could be included in our review. The answers were negative. The three studies were: PI Johansson and colleagues, total 832 cases, 121 traumas, raw data unavailable for trauma [1]; BM Messenger and colleagues, prospective study, 50 cases, mortality similar, raw data unavailable [2]; and K Kashuk and colleagues, prospective study, 68 cases, mortality 59% control group, 28% VHT group [3].

## Conclusion

With the studies available as of the end of 2014, it is impossible to conclude whether the use of a VHT in the emergency department decreases mortality. Other studies are needed.
